# Influence of Asymmetry and Driving Forces on the Propulsion of Bubble-Propelled Catalytic Micromotors

**DOI:** 10.3390/mi7120229

**Published:** 2016-12-14

**Authors:** Masayuki Hayakawa, Hiroaki Onoe, Ken H. Nagai, Masahiro Takinoue

**Affiliations:** 1 Department of Computational Intelligence and Systems Science, Tokyo Institute of Technology, Yokohama, Kanagawa 226-8502, Japan; hayakawa.m.ac@m.titech.ac.jp; 2 Department of Mechanical Engineering, Keio University, Yokohama, Kanagawa 223-8522, Japan; onoe@mech.keio.ac.jp; 3 School of Materials Science, Japan Advanced Institute of Science and Technology, Nomi, Ishikawa 923-1292, Japan; k.nagai0325@gmail.com; 4 Department of Computer Science, School of Computing, Tokyo Institute of Technology, Yokohama, Kanagawa 226-8502, Japan

**Keywords:** self-propelled micromotors, bubble propulsion, complex-shaped multi-compartmental microparticles, complex-shaped multi-compartmental microgels, active matter

## Abstract

Bubble-propelled catalytic micromotors have recently been attracting much attention. A bubble-propulsion mechanism has the advantage of producing a stronger force and higher speed than other mechanisms for catalytic micromotors, but the nature of the fluctuated bubble generation process affects the motions of the micromotors, making it difficult to control their motions. Thus, understanding of the influence of fluctuating bubble propulsion on the motions of catalytic micromotors is important in exploiting the advantages of bubble-propelled micromotors. Here, we report experimental demonstrations of the bubble-propelled motions of propeller-shaped micromotors and numerical analyses of the influence of fluctuating bubble propulsion on the motions of propeller-shaped micromotors. We found that motions such as trochoid-like motion and circular motion emerged depending on the magnitude or symmetricity of fluctuations in the bubble-propulsion process. We hope that those results will help in the construction and application of sophisticated bubble-propelled micromotors in the future.

## 1. Introduction

Self-propelled catalytic micromotors operating at a low Reynolds number, which are powered by chemical reactions, have attracted significant interest not only in the fields of non-equilibrium science about chemical-to-mechanical energy transduction [1,2,3,4,5,6,7] but also in nano/microengineering for the construction of autonomous micromotors [8,9,10,11,12,13,14,15]. Well-known mechanisms of self-propelled catalytic micromotors are the self-diffusiophoresis mechanism and the bubble propulsion mechanism. In the self-diffusiophoresis mechanism [16], asymmetrically allocated catalysts on the surface of a micromotor decompose substrate molecules around the micromotor and generate a gradient of decomposition products around the micromotor. As a result, an osmotic force due to the product gradient propels the micromotor in the direction of the gradient. Recent studies have shown the influence of various parameters such as the allocation of catalytic sites [17] and surface roughness of micromotors [18] on their motion [17,18,19,20,21]. On the other hand, in the bubble propulsion mechanism, a micromotor with asymmetrically allocated catalysts is driven by repetitive bubble growth [22,23] and detachment [24]. According to previous studies, the bubble propulsion mechanism can provide higher velocity and stronger force than the self-diffusiophoresis mechanism [23,25]. These features of the bubble propulsion mechanism are advantageous for various situations such as the propulsion of micromotors against a flow [26], transportation of large cargo [27,28], and drilling a cell [29]. Fundamental studies of bubble propulsion mechanisms [22,23,30,31,32,33], such as fluid transport induced by bubble-propelled micromotors [32], have also been conducted. However, it is still difficult to precisely control the motion of micromotors. This is because the nucleation, growth and detachment of bubbles randomly occur at several points on the catalytic surfaces [30]; additionally, the bubble size and the bubble production rate are affected by many factors such as local surface tension [31], chemical reaction efficiencies [31], and surface roughness [23,33,34]. Since the strength of the pushing force of bubbles is proportional to the size of the bubbles [24,35], the fluctuation of the bubble size results in the fluctuation of the force strength. Thus, understanding the self-propulsions with the force strength fluctuations due to the random bubble propulsion process is important in order to exploit the advantages of bubble-propelled micromotors. A study of motions of complex-shaped micromotors under the fluctuation of force strength is especially important because the shapes of the micromotors influence the bubble generation process [35] and predominantly determine their motion and velocity.

In this study, we report on the bubble-propelled motions of propeller-shaped micromotors with a single catalytic site and double catalytic sites. First, we show the construction of the propeller-shaped micromotors and experimentally demonstrate the bubble-propelled motions of the micromotors. Next, the bubble-propelled motions of the propeller-shaped micromotors with driving force fluctuation are numerically analysed using overdamped equations of motion [17,36]. We show that motions depend on the magnitude or symmetricity of the fluctuation of force strength. We believe that this study will aid in the use of bubble-propelled micromotors with various complex shapes in noisy circumstances such as human bodies, natural environments, and artificial structures such as microfluidic channels.

## 2. Materials and Methods

### 2.1. Materials and Experimental Setup

**Figure 1:**
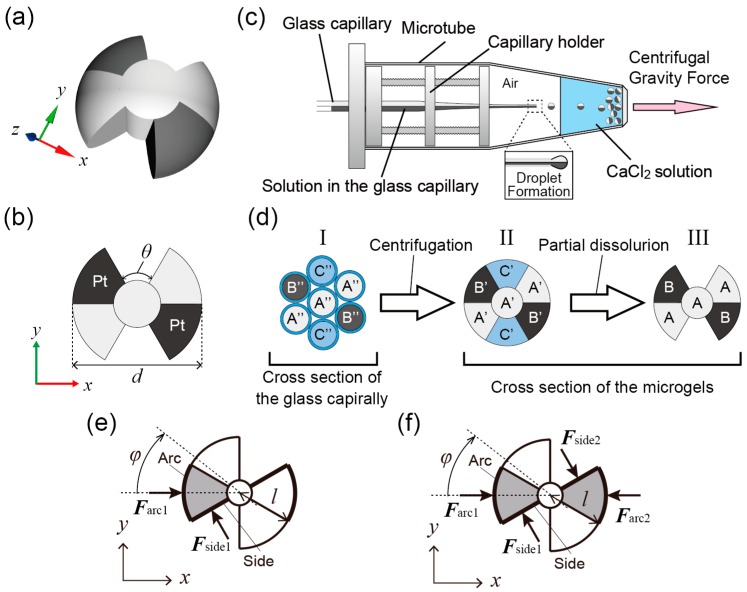
Schematic illustrations of the propeller-shaped micromotor and synthesizing methods. (**a**) 3D sketch of the micromotor; (**b**) 2D sketch of the micromotor with diameter *d* ~140 μm and an angle of propeller θ ~50°; (**c**) Schematic illustrations of centrifuge-based droplet-shooting device (CDSD); (**d**) Synthesizing diagram of the propeller-shaped micromotors. A cross sectional image of the glass capillary (I), the spherical microparticles (II) and the propeller-shaped micromotors with PtNPs (III) are shown; (**e**,**f**) Designs of a propeller-shaped micromotor used in our experiments and numerical simulation with a single catalytic site (**e**) and with double catalytic sites (**f**).

We constructed bubble-propelled micromotors (Figure 1a,b) using agarose gel microparticles with multi-compartmentalized propeller-shaped structures [37]. The micromotors were produced using a centrifuge-based droplet-shooting device (CDSD) [38]. The CDSD consisted of a septuple-barreled glass capillary (World Precision Instruments, 7B100F-6, Sarasota, FL, USA) containing mixture polymer solutions of a sodium alginate solution (Wako Pure Chemical Industries, Osaka, Japan), a capillary holder (Figure S1), and a sampling microtube (BIO-BIK, 1.5 mL microtube CF-0150, Osaka, Japan) containing a 0.5 M CaCl_2_ solution (Wako Pure Chemical Industries) (Figure 1c). Figure 1d shows the synthesizing process for the propeller-shaped micromotor with double catalytic sites. First, three types of polymer solutions were introduced into different compartments of the septuple-barreled glass capillary (Figure 1d-I). These solutions were a mixture solution of 2% (*w*/*w*) agarose (Sigma-Aldrich, Type IX-A Ultra-low Gelling Temperature, A2576, St. Louis, MO, USA) and 2% (*w*/*w*) sodium alginate with 0.5% (*w*/*w*) 1 μm polystyrene nanoparticles (PSNPs) (Polysciences, Polybead carboxylate 1.0 micron microspheres, 07310, Warminster, PA, USA) in the A″ part, a mixture solution of agarose and sodium alginate with platinum nanoparticles (PtNPs) (Sigma-Aldrich, platinum powder, ≤10 µm, 327476) in the B″ part, and 3% (*w*/*w*) sodium alginate solution in the C″ part. The PSNPs were used only for microscopic observations and the PtNPs were used as catalysts. In addition, all mixture solutions contained 0.1% (*w*/*w*) TritonX-100 (Wako Pure Chemical Industries) to prevent aggregations of the PtNPs and the PSNPs, and to balance surface tension [37]. Through the centrifugation of the CDSD, all polymer solutions were dripped from the tip of the capillary (Figure 1c). Then, the detached droplet was solidified at the bottom of the microtube because of the gelation of the sodium alginate solutions in all compartments. After the centrifugation, the droplets were cooled at ~4 °C for ~20 min. The obtained multi-compartmental gel microparticles (Figure 1d-II) consisted of three parts: A′, an interpenetrating network (IPN) gel of a calcium alginate and an agarose gel with the PSNPs; B′, the IPN gel of the calcium alginate and the agarose gel with the PtNPs; and C′, the calcium alginate gel. The calcium alginate gel in all parts was dissolved away by removing Ca^2+^ ions with a calcium-chelating agent, ethylenediamine-tetraacetic acid (EDTA) (Wako Pure Chemical Industries). The final concentration of the EDTA was 0.25 M. As a result, the propeller-shaped micromotors with PtNPs were produced; as shown in Figure 1d-III, they consisted of agarose gel with the PSNPs (A part) and agarose gel with the PtNPs (B part).

The dispersion solution of the propeller-shaped micromotors (~0.5 μL) was put into a glass petri dish filled with 10 mL of an aqueous solution including 15% (*w*/*w*) H_2_O_2_ (Wako Pure Chemical Industries), 0.0005% (*w*/*w*) benzalkonium chloride (Wako Pure Chemical Industries), and 1% (*v*/*v*) isopropanol (Wako Pure Chemical Industries) (Figure S2a). The catalyst, PtNPs, in the micromotors decomposed hydrogen peroxide molecules in the solution and generated oxygen bubbles: 2H_2_O_2_ $\overset{\mathrm{Pt}}{\to }$ 2H_2_O + O_2_. The motions of micromotors were observed using a digital microscope (KEYENCE, VHX-2000, Osaka, Japan) (Figure S2a). Trajectories of the micromotors were manually tracked in steps of 0.2 s using Image J (National Institutes of Health, New York, NY, USA, 2015, 1.50a). To exclude the effects of an unintended flow of surrounding H_2_O_2_ solution, the tracked coordinate of a floating bubble irrelevant to motor propulsion was subtracted from the tracked coordinate of the self-propelled micromotor.

### 2.2. Numerical Simulation and Numerical Model

When a generated O_2_ bubble is detached from the catalytic surface of a micromotor, a pushing force acts on the surface [24]. The motions of the micromotors are considered in a two-dimensional plane (*xy*-plane). The bubble-propelled motion of the propeller-shaped micromotor with a single catalytic site (Figure 1e) is described by the following over-damped equations of motion for translation and for rotation:

(1) $$\eta_{\mathrm{tra}}\frac{dr}{dt}=F_{1}\;,$$

(2) $$\eta_{\mathrm{rot}}\frac{d\varphi }{dt}=\frac{l}{2}F_{1\mathrm{rot}}\;,$$

where ***r***(*t*) = (*x*(*t*), *y*(*t*)) is the position of a micromotor (the initial position: ***r***(0) = (0, 0)); φ(t) is the rotational angle of the micromotor (φ(0)=0); *t* is time; ***F***_1_ (=***F***_side1_ + ***F***_arc1_) is the net pushing force from the catalytic surfaces; ***F***_side1_ and ***F***_arc1_ are the pushing forces from the side and the arc of the blade, respectively; *F*_1rot_ is the rotation-direction component of ***F***_1_; *l* (~7.0 × 10^−5^ m) is the arm length; η_tra_ (~1.3 × 10^−6^ kg·s^−1^) and η_rot_ (~8.5 × 10^−15^ m^2^·kg·s^−1^) denote coefficients of viscous resistance for translation and rotation, respectively. The force strengths of ***F***_side1_ and ***F***_arc1_ have fluctuation as |***F***_side1_| = *f*_side1_ + ξ_side1_ and |***F***_arc1_| = *f*_arc1_ + ξ_arc1_. *f*_side1_ (=2 × 10^−10^ N) and *f*_arc1_ (=0.75 *f*_side1_) are constant. ξ_side1_ and ξ_arc1_ are Gaussian noises, where the means of ξ_side1_ and ξ_arc1_ are 0, and their standard deviations are σ_side1_ and σ_arc1_, respectively. We define normalized magnitude of fluctuation as $\hat{\sigma }$_side1_ = σ_side1_*/*σ_0_ and $\hat{\sigma }$_arc1_ = σ_arc1_*/*σ_0_ (normalization constant σ_0_ = 3 × 10^−11^ N). When *f*_side1_ + ξ_side1_ < 0, we set |***F***_side1_| = 0. Similarly, when *f*_arc1_ + ξ_arc1_ < 0, we set |***F***_arc1_| = 0. When σ_side1_ is small (~0 N), an approximately steady force |***F***_side1_| ~ *f*_side1_ acts on the micromotor. This corresponds to the situation in which generated bubble size is relatively uniform. When σ_side1_ is large (>*f*_side1_), |***F***_side1_| often has the force value ‘0’ and sometimes has a large force value (>2 *f*_side1_) (Figure S3). The value ‘0’ corresponds to the waiting time to grow a large bubble, and the large value corresponds to the large force due to the large bubble sometimes generated, because a larger bubble generates a stronger force but requires a longer waiting time to grow until it reaches the detachment radius [24]. These situations are the same in the case of |***F***_arc1_| (Figure S3). All numerical calculations were carried out for 2000 steps with a step interval of 0.05 s using the numerical computation software MATLAB (MathWorks Inc., Natick, MA, USA).

The bubble-propelled motion of the propeller-shaped micromotor with double catalytic sites (Figure 1f) is similarly described by the following over-damped equations of motion for translation and for rotation:

(3) $$\eta_{\mathrm{tra}}\frac{dr}{dt}=F_{1}+F_{2}\;,$$

(4) $$\eta_{\mathrm{rot}}\frac{d\varphi }{dt}=\frac{l}{2}(F_{1\mathrm{rot}}+F_{2\mathrm{rot}}),$$

where ***F***_2_ (=***F***_side2_ + ***F***_arc2_) is the net pushing force from the catalytic surfaces; *F*_2rot_ is the rotation-direction component of ***F***_2_; ***F***_side2_ (|***F***_side2_| = *f*_side2_ + ξ_side2_, *f*_side2_ = *f*_side1_) and ***F***_arc2_ (|***F***_arc2_| = *f*_arc2_ + ξ_arc2_, *f*_arc2_ = *f*_arc1_) are the pushing forces from the side and the arc of the blade, respectively. ξ_side2_ and ξ_arc2_ are Gaussian noises: their means are 0, and their standard deviations are σ_side2_ and σ_arc2_, respectively. We define a set of normalized magnitudes of fluctuation Σ = ($\hat{\sigma }$_side1_, $\hat{\sigma }$_arc1_, $\hat{\sigma }$_side2_, $\hat{\sigma }$_arc2_) = (σ_side1_*/*σ_0_, σ_arc1_*/*σ_0_, σ_side2_*/*σ_0_, σ_arc2_*/*σ_0_). All numerical calculations were carried out for 2000 steps with a step interval of 0.05 s using MATLAB.

For statistical analyses, mean square displacement (MSD) $G_{\mathrm{MSD}}\left(\Delta t\right)$ is defined as follows:

(5) $$G_{\mathrm{MSD}}\left(\Delta t\right)=\;\frac{1}{N\left(T-\Delta t\right)}\overset{N}{\underset{i=1}{\sum }}\overset{T-\Delta t}{\underset{0}{\int }}{\left\{r_{i}\left(t+\Delta t\right)-r_{i}\left(t\right)\right\}}^{2}dt,$$

where *N* is the number of micromotors; *T* is the final time of the simulations. In this study, *N* = 300 and *T* = 100 s.

## 3. Results and Discussion

### 3.1. Experimental Observation of Bubble-Propelled Motions of Propeller-Shaped Micromotors

**Figure 2:**
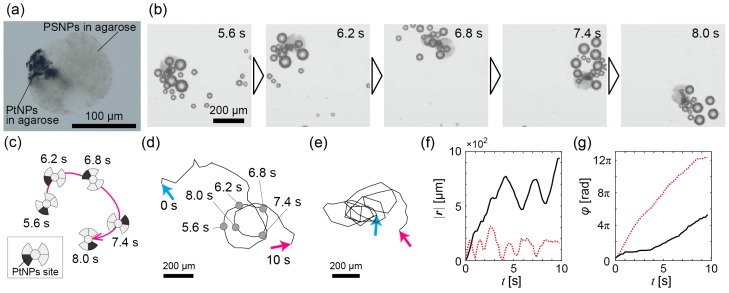
Experimental results of bubble propulsion of the propeller-shaped micromotors with a single catalytic site in the H_2_O_2_ solution. (**a**) A microscope image of a propeller-shaped micromotor with a single catalytic site; (**b**) Time series from *t* = 5.6 s to *t* = 8.0 s of a propeller-shaped micromotor with a single catalytic site propelled by bubbles; (**c**) Schematic illustration of the trajectory of (**b**); (**d**) The whole trajectory of the micromotor in (**b**); Cyan arrow: *t* = 0 s; magenta arrow: *t* = 10 s; (**e**) Trajectory of another micromotor. The notation of arrows is the same as in (**d**); (**f**) The time variation of |***r***(*t*)|. Black solid line: For trajectory of (**d**); red dashed line: For trajectory of (**e**); (**g**) The time variation of φ(t). The notation of each line is the same as in (**f**).

Figure 2a is a microscope image of a propeller-shaped micromotor with a single catalytic site (*d* ~140 μm and θ ~50°; Figure S4a,c,e), which suggests that our method [37] can be used to construct various complex-shaped multi-compartmental bubble-propelled micromotors. In the H_2_O_2_ solution, the micromotors were spontaneously propelled near an interface of the solution due to generated bubbles (Figure S2b,c). Figure 2b shows time-series images of the bubble propulsion of the micromotor motion; its schematic illustration is shown in Figure 2c. The micromotor produced various-sized bubbles and was propelled by those bubbles. Figure 2d shows the whole trajectory of the micromotor of Figure 2b (see also Supplementary Video S1). A trajectory of another micromotor is shown in Figure 2e (see also Supplementary Video S2). Both trajectories show circular trajectories, but they are perturbed and not true circles. |***r***(*t*)| and φ(t) of the trajectory of both the micromotors are shown in Figure 2f,g. The time courses of |***r***(*t*)| in Figure 2f suggest that both the micromotors had about 1–2 s of circular periods, although the periods were perturbed. In addition, φ(t) time courses in Figure 2g indicate that the circular motions were accompanied by monotonic rotations of the micromotors.

**Figure 3:**
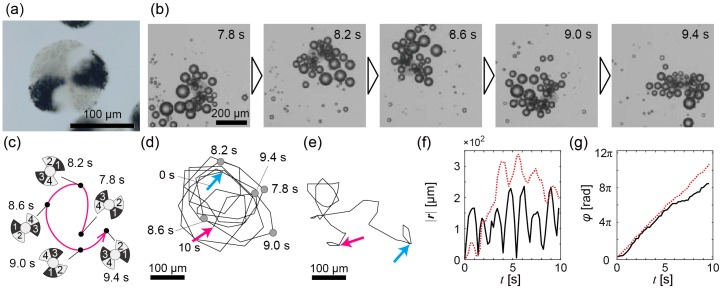
Experimental results of bubble propulsion of the propeller-shaped micromotors with double catalytic sites in the H_2_O_2_ solution. (**a**) A microscope image of a propeller-shaped micromotor with double catalytic sites; (**b**) Time series from *t* = 7.8 s to *t* = 9.4 s of a propeller-shaped micromotor with double catalytic sites propelled by bubbles; (**c**) Schematic illustration of the trajectory of (**b**); (**d**) The whole trajectory of the micromotor in (**b**); Cyan arrow: *t* = 0 s; magenta arrow: *t* = 10 s; (**e**) Trajectory of another micromotor. The notation of arrows is the same as in (**d**); (**f**) The time variation of |***r***(*t*)|. Black solid line: For trajectory of (**d**); red dashed line: For trajectory of (**e**); (**g**) The time variation of φ(t). The notation of each line is the same as in (**f**).

Figure 3a is a microscope image of a propeller-shaped micromotor with double catalytic sites (*d* ~140 μm and θ ~50°; Figure S4b,d,f). The two catalytic sites were symmetrically allocated at the opposite blades. Like this example, change of catalytic sites is easy for our construction method of bubble-propelled propeller-shaped micromotors [37]. Similarly, the micromotors were propelled near an interface due to generated bubbles (Figure S2b,c). Figure 3b presents time-series images of the bubble propulsion of a micromotor, and Figure 3c is a schematic illustration of Figure 3b. Its entire trajectory is shown in Figure 3d (see also Supplementary Video S3). A trajectory of another micromotor is shown in Figure 3e (see also Supplementary Video S4). |***r***(*t*)| and φ(t) of the trajectory of both the micromotors are shown in Figure 3f,g. Since the catalytic sites were allocated symmetrically, the rotations of the micromotors were observed as expected (Figure 3g). In addition, the fluctuated translational motions were also observed (Figure 3f); the case of the black solid line of Figure 3f seems to be a perturbed circular motion with a period of about 1 s.

### 3.2. Numerical Analyses of Bubble-Propelled Motions of Propeller-Shaped Micromotors

The perturbed motions observed in the experiments are considered to be due to the fluctuations in produced bubble size as well as the experimental inaccuracy of the construction of micromotors. In order to investigate the bubble-propelled motions of propeller-shaped micromotors more generally, numerical simulations and analyses were performed. We focus especially on the influence of the force strength fluctuation induced by the fluctuation of bubble generation on the bubble-propelled motions of propeller-shaped micromotors.

**Figure 4:**
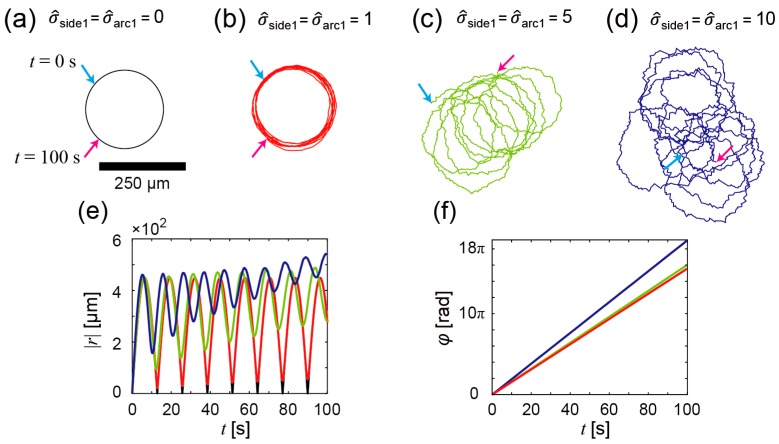
Numerical analyses of bubble propulsion of the propeller-shaped micromotors with a single catalytic site. (**a**–**d**) Calculated trajectories of the micromotor in each condition of $\hat{\sigma }$_side1_ and $\hat{\sigma }$_arc1_. $\hat{\sigma }$_side1_ = $\hat{\sigma }$_arc1_ = 0 (**a**); $\hat{\sigma }$_side1_ = $\hat{\sigma }$_arc1_ = 1 (**b**); $\hat{\sigma }$_side1_ = $\hat{\sigma }$_arc1_ = 5 (**c**); $\hat{\sigma }$_side1_ = $\hat{\sigma }$_arc1_ = 10 (**d**); Cyan arrow: *t* = 0 s; magenta arrow: *t* = 100 s; (**e**) The time variation of |***r***(*t*)|. Black line: For trajectory of (**a**); red line: For trajectory of (**b**); green line: For trajectory of (**c**); blue line: For trajectory of (**d**); (**f**) The time variation of φ(t). The notation of each line is the same as in (**e**).

First, we numerically calculated trajectories of the propeller-shaped micromotors with a single catalytic site (Figure 1e) using Equations (1) and (2); the results are shown in Figure 4a–d. Figure 4a shows a trajectory of the micromotor without the fluctuation of the force strength ($\hat{\sigma }$_side1_ = $\hat{\sigma }$_arc1_ = 0). Since no fluctuation disturbs the motions of the micromotor, the trajectory exhibits a true circle, which is due to the asymmetric allocation of the catalytic sites. As $\hat{\sigma }$_side1_ and $\hat{\sigma }$_arc1_ increase, the trajectories are perturbed by the fluctuation of the force strength: $\hat{\sigma }$_side1_ = $\hat{\sigma }$_arc1_ = 1 (Figure 4b); $\hat{\sigma }$_side1_ = $\hat{\sigma }$_arc1_ = 5 (Figure 4c); $\hat{\sigma }$_side1_ = $\hat{\sigma }$_arc1_ = 10 (Figure 4d). Figure 4e,f show the time courses of |***r***(*t*)| and φ(t). Periodic increase and decrease in |***r***(*t*)| is observed when the fluctuation of the force strength is small; as the fluctuation increases, the increase of the basal value of |***r***(*t*)| in addition to the periodic increase and decrease is observed, which indicates that translational displacements of the micromotors occur. Periodic rotations of the micromotors are also observed (Figure 4f). From these results, we guess that the experimentally observed perturbed trajectories of the micromotors (Figure 2d,e) were induced by the fluctuations in the force strength.

**Figure 5:**
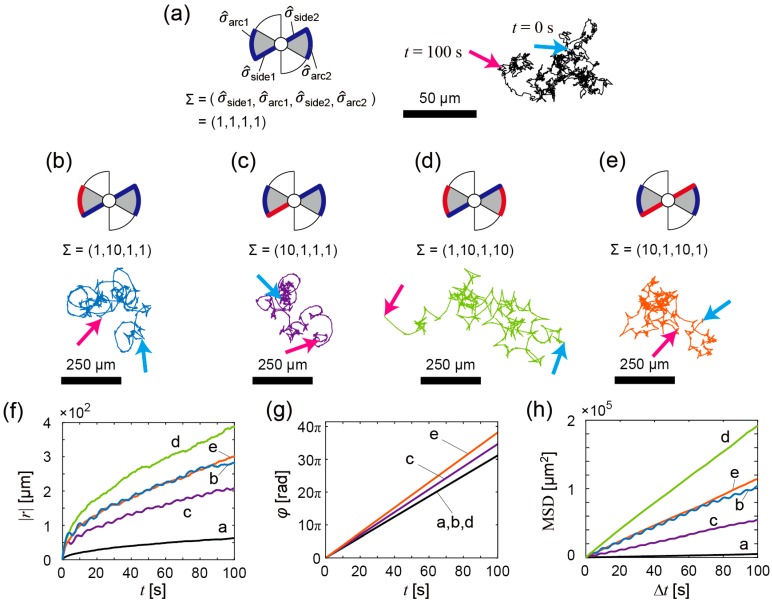
Numerical analyses of bubble propulsion of the propeller-shaped micromotors with double catalytic sites. (**a**–**e**) Calculated trajectories of the micromotor in each condition of Σ = ($\hat{\sigma }$_side1_,$\hat{\sigma }$_arc1_,$\hat{\sigma }$_side2_,$\hat{\sigma }$_arc2_). Red lines/curves: the sides and the arcs that generate a pushing force with large fluctuation. Blue lines/curves: the sides and the arcs that generate a pushing force with small fluctuation. Σ = (1, 1, 1, 1) (symmetric) (**a**); Σ = (1, 10, 1, 1) (asymmetric) (**b**); Σ = (10, 1, 1, 1) (asymmetric) (**c**); Σ = (1, 10, 1, 10) (symmetric) (**d**); Σ = (10, 1, 10, 1) (symmetric) (**e**); Cyan arrow: *t* = 0 s; magenta arrow: *t* = 100 s; (**f**) The time variation of |***r***(*t*)|. Black line: For trajectory of (**a**); blue line: For trajectory of (**b**); purple line: For trajectory of (**c**); green line: For trajectory of (**d**); orange line: For trajectory of (**e**); (**g**) The time variation of φ(t). The notation of each line is same as (**f**); (**h**) The time variation of mean square displacement (MSD) $G_{\mathrm{MSD}}\left(t\right)$. The notation of each line is same as (**f**).

Next, we numerically calculated trajectories of the propeller-shaped micromotors with double catalytic sites (Figure 1f) using Equations (3) and (4). Figure 5a–e show calculated trajectories of micromotors with various fluctuations (i.e., various Σ values) of pushing forces. The basal values of pushing forces are translationally and rotationally balanced in all cases (i.e., *f*_side1_ = *f*_side2_ and *f*_arc1_ = *f*_arc2_) because the catalytic sites are allocated symmetrically, but the magnitudes of fluctuation of the pushing forces are not identical. In Figure 5a–e, the sides and the arcs that generate a pushing force with large fluctuation are drawn with red lines/curves, and those that generate a pushing force with small fluctuation are drawn with blue lines/curves. The motion patterns vary depending on Σ values as shown by the trajectory in Figure 5a–e and |***r***(*t*)| in Figure 5f, but the micromotors in all cases periodically rotate as shown by φ(t) in Figure 5g. In the cases of Figure 5a,d,e, the magnitude of fluctuation of pushing forces are translationally and rotationally symmetric. Thus, the bubble-propelled motions of micromotors seem to be approximately random motions as confirmed by the linear relationship between MSD and Δ*t* (Figure 5h). In the case of Figure 5b, the magnitude of fluctuation of pushing forces is translationally asymmetric; as a result, a circular motion emerges due to the fluctuation asymmetricity even though the basal values of pushing forces are translationally and rotationally balanced. The circular motion is observed as a periodic change of |***r***(*t*)| (Figure 5f) and $G_{MSD}\left(\Delta t\right)$ (Figure 5h). In the case of Figure 5c, the magnitudes of fluctuations in pushing forces are rotationally asymmetric; this case also shows the emergence of a circular motion due to the fluctuation asymmetricity. In the cases of Figure 5a,d,e, $G_{MSD}\left(\Delta t\right)$ (Figure 5h) shows approximate linear time variation, which indicates that the motion is approximately random. On the other hand, in the cases of Figure 5b,c, $G_{MSD}\left(\Delta t\right)$ shows time variation with periodic increase and decrease (Figure 5h), which indicates that the circular motions emerge as statistically predominant. The emergent circular motion (Figure 5b) corresponds to the experimental data as shown in Figure 3d,e.

## 4. Conclusions

In conclusion, we constructed propeller-shaped micromotors with a single catalytic site and double catalytic sites and showed experimental demonstrations of the bubble-propelled motions of both the propeller-shaped micromotors. We then numerically investigated the motions in terms of the influence of the bubble size fluctuation on the motions of the propeller-shaped micromotors, where the magnitude of force fluctuation was changed instead of the bubble-size fluctuation. In the propeller-shaped micromotors with both single catalytic and double catalytic sites, the experimentally observed trajectories of the micromotor were perturbed circular motions with rotations (Figure 2 and Figure 3). In the numerical analyses, we first investigated the propeller-shaped micromotors with a single catalytic site, and showed that the trajectories become perturbed circular motions with translational displacement (Figure 4), similar to a trochoid-like motion. Next, we investigated the propeller-shaped micromotors with double catalytic sites and, as a result, we found that circular motions emerge due to the asymmetry of the magnitudes of fluctuations (Figure 5) in pushing forces acting on the propeller blades.

This study will provide useful insights into not only the propeller-shaped micromotor we showed, but also other complex-shaped micromotors. Because the motion of a rigid body can be divided into translational and rotational components, in any case of micromotor shapes, we can separately consider the asymmetricity of fluctuations of translational and rotational components. For example, the knowledge obtained from this study can be utilized for the design of bubble-propelled chemotactic micromotors that can move from an initial point to a goal. Assuming that there exists less surfactant at the initial point and more at the goal, a propeller-shaped micromotor with a single catalytic site (Figure 1e) exhibits a fluctuated trochoid-like motion (Figure 4c,d) near the initial point but exhibits a true circular motion (Figure 4b) near the goal because the bubble size gets more uniform (i.e., the bubble size fluctuation reduces) when the local surface tension decreases with increase in the surfactant [34]. As a result, the micromotor randomly moves near the initial point, and then moves to the direction of the goal by chance; finally, the micromotor continues a stable true circular motion around the goal. This idea is based on the positive use of the difference in the fluctuated trochoid-like motion and stable circular motion to realize chemotaxis.

The circular motions (Figure 5b,c) are nontrivial enhanced motions in bubble propulsion due to the asymmetry of force fluctuations induced by the fluctuations in bubble generation. In self-diffusiophoresis, the fluctuation of force strength is small and additionally symmetric because the catalytic reaction occurs uniformly throughout the catalyst surface. Our study will promote strategies to enhance motions of bubble-propelled micromotors with useful functions [26,27,28,29]. For example, using the non-uniform concentration field near boundaries such as a wall of microfluidic channels [20], a blood vessel [39], and a stomach [40], there are possibilities for micromotors to change their own motions. We hope that our experimental and numerical results will promote the construction of a sophisticated bubble-propelled micromotor.

## Supplementary Materials

The following are available online at www.mdpi.com/2072-666X/7/12/229/s1. Figure S1: Fabrication of the capillary holder; Figure S2: Observation of the micromotors; Figure S3: Histograms of the force generated from the side and the arc; Figure S4: Measurements of size and shapes of the propeller-shaped micromotors, Videos S1 and S2: The motions of the propeller-shaped micromotors with a single catalytic site, Vides S3 and S4: The motions of the propeller-shaped micromotors with double catalytic sites.
