# Erratum: Hayakawa, M., et al. Influence of Asymmetry and Driving Forces on the Propulsion of Bubble-Propelled Catalytic Micromotors. *Micromachines* 2016, *7*, 229

**DOI:** 10.3390/mi8020061

**Published:** 2017-02-20

**Authors:** Micromachines Editorial Office

**Affiliations:** MDPI AG, St. Alban-Anlage 66, 4052 Basel, Switzerland; micromachines@mdpi.com

The editorial team of the journal *Micromachines* would like to make the following changes to the published paper [1]: The captions of Figure 2 and Figure 3 are correct, however, the images of Figure 2 and Figure 3 should be exchanged as follows:

We apologize for any inconvenience caused to the readers or authors by this error. The change does not affect the scientific results. The article will be updated and the original will remain on the article webpage.

## Figures and Tables

**Figure 2:**
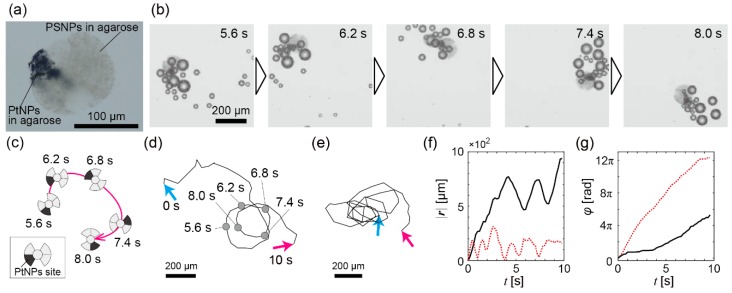
Experimental results of bubble propulsion of the propeller-shaped micromotors with a single catalytic site in the H_2_O_2_ solution. (**a**) A microscope image of a propeller-shaped micromotor with a single catalytic site; (**b**) Time series from *t* = 5.6 s to *t* = 8.0 s of a propeller-shaped micromotor with a single catalytic site propelled by bubbles; (**c**) Schematic illustration of the trajectory of (**b**); (**d**) The whole trajectory of the micromotor in (**b**); Cyan arrow: *t* = 0 s; magenta arrow: *t* = 10 s; (**e**) The trajectory of another micromotor. The notation of arrows is the same as in (**d**); (**f**) The time variation of |***r***(*t*)|. Black solid line: For the trajectory of (**d**); red dashed line: For the trajectory of (**e**); (**g**) The time variation of φ(t). The notation of each line is the same as in (**f**).

**Figure 3:**
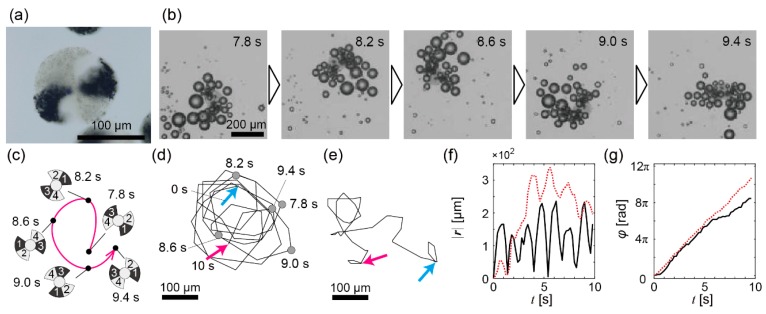
Experimental results of bubble propulsion of the propeller-shaped micromotors with double catalytic sites in the H_2_O_2_ solution. (**a**) A microscope image of a propeller-shaped micromotor with double catalytic sites; (**b**) Time series from *t* = 7.8 s to *t* = 9.4 s of a propeller-shaped micromotor with double catalytic sites propelled by bubbles; (**c**) Schematic illustration of the trajectory of (**b**); (**d**) The whole trajectory of the micromotor in (**b**); Cyan arrow: *t* = 0 s; magenta arrow: *t* = 10 s; (**e**) The trajectory of another micromotor. The notation of arrows is the same as in (**d**); (**f**) The time variation of |***r***(*t*)|. Black solid line: For the trajectory of (**d**); red dashed line: For the trajectory of (**e**); (**g**) The time variation of φ(t). The notation of each line is the same as in (**f**).
