# Supplementary material for: Influence of Asymmetry and Driving Forces on the Propulsion of Bubble-Propelled Catalytic Micromotors
Source: Micromachines (Basel). 2016 Dec 14;7(12):229. doi: 10.3390/mi7120229 (PMC6190221; doi:10.3390/mi7120229)
Supplement: Supplementary file 1 [file micromachines-07-00229-s001.zip › micromachines-157973-Supple-to be published.pdf]

Supplementary Materials: Influence of Asymmetry and Driving Forces on the Propulsion of Bubble-Propelled Catalytic Micromotors

Masayuki Hayakawa, Hiroaki Onoe, Ken H. Nagai and Masahiro Takinoue

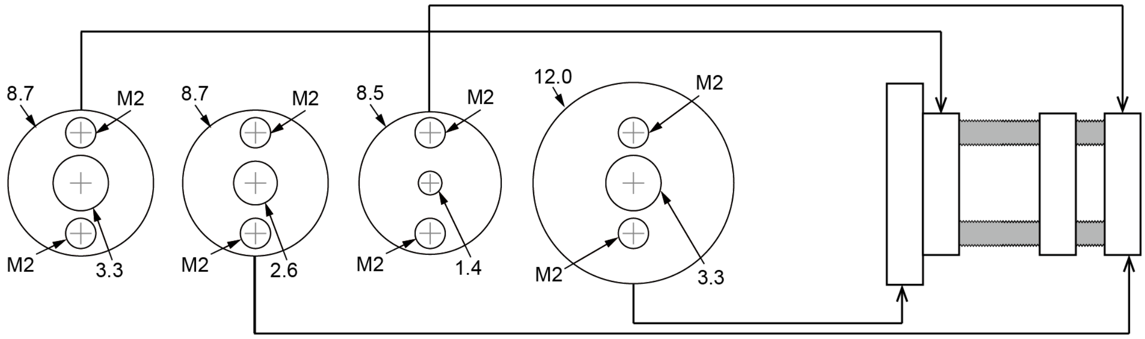

Figure S1. Fabrication of the capillary holder. A schematic illustration of a capillary holder. Using a MODERA MDX-40 (Roland DG, Hamamatsu, Japan), polyacetal resin plates (2 mm thickness) were cut into circular plates, and fixing holes for the capillary and screw holes were drilled. The processed plates were assembled with screws (M2 × 1.5) and finally, the screw heads were removed.

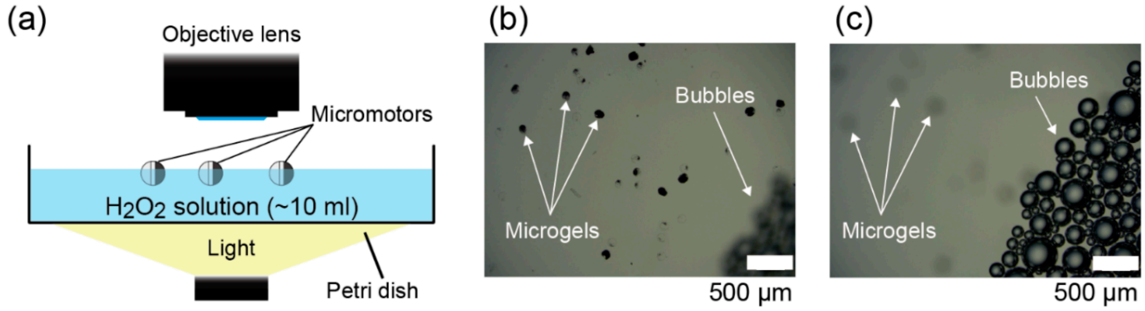

Figure S2. Observation of the micromotors. The experimental set up for the observation of motions of the micromotors is shown in (**a**). The petri dish was filled with 10 mL of an aqueous solution including 15% (*w*/*w*) H_2O_2 (Wako Pure Chemical Industries, Osaka, Japan), 0.0005% (*w*/*w*) benzalkonium chloride (Wako Pure Chemical Industries), and 1% (*v*/*v*) isopropanol (Wako Pure Chemical Industries). The motions of micromotors were observed using a digital microscope (KEYENCE, VHX-2000, Osaka, Japan); (**b**) Microscope image focused on microgels at the bottom of the petri dish; and (**c**) focused on floating bubbles on the surface of the solution. In this experiment, the petri dish was filled with 10 mL of aqueous solution including 0.0005% (*w*/*w*) benzalkonium chloride and 1% (*v*/*v*) isopropanol; H_2O_2 was not included to avoid the bubble generations by the PtNPs in the microgels. The image shown in (**b**) focused on microgels sinking to the bottom without bubble generation, and did not focus on bubbles floating on the surface at all. On the other hand, the picture shown in (**c**) focused on the bubbles floating on the surface, and did not focus on microgels sinking to the bottom at all. Thus, these images suggest that the micromotors propelled near the interface due to the buoyancy of the generated bubble.

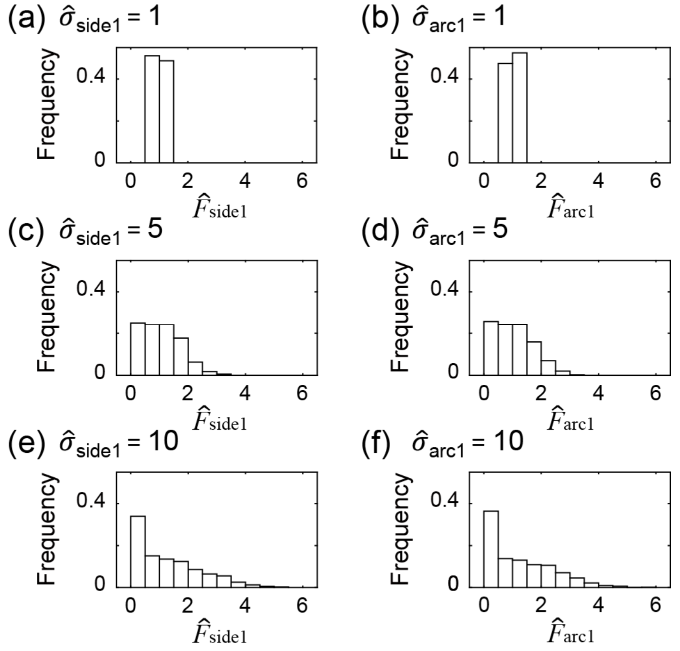

Figure S3. Histograms of the force generated from the side and the arc. The histograms of normalized forces $\hat{F}_{side1} = (f_{side} + \xi_{side1})/f_{side}$ and $\hat{F}_{arc1} = (f_{arc} + \xi_{arc1})/f_{arc}$ are shown. As mentioned in the main text, we define normalized standard deviation, $\hat{\sigma}_{side1} = \sigma_{side1}/\sigma_0$ and $\hat{\sigma}_{arc1} = \sigma_{arc1}/\sigma_0$. When $\hat{\sigma}_{side1}$ and $\hat{\sigma}_{side1}$ are small (**a**,**b**), $\hat{F}_{side1} \sim 1$ and $\hat{F}_{arc1} \sim 1$ ($|\boldsymbol{F}_{side1}| \sim f_{side1}$ and $|\boldsymbol{F}_{arc1}| \sim f_{arc1}$). This condition corresponds to the situation in which the micromotor generates uniform-sized bubbles and causes less fluctuation of driving forces. As $\hat{\sigma}_{side1}$ and $\hat{\sigma}_{arc1}$ increase (**c**–**f**), $\hat{F}_{side1} = 0$ and $\hat{F}_{arc1} = 0$ ($|\boldsymbol{F}_{side1}| = 0$ and $|\boldsymbol{F}_{arc1}| = 0$) are sometimes generated. The value '0' corresponds to the waiting time to grow a large bubble. On the other hand, when σ_{side1} and σ_{arc1} are large, larger forces $\hat{F}_{side1} > 2$ and $\hat{F}_{arc1} > 2$ ($|\boldsymbol{F}_{side1}| > 2f_{side}$ and $|\boldsymbol{F}_{arc1}| > 2f_{arc}$) are also generated. These larger forces correspond to the forces induced by large bubbles.

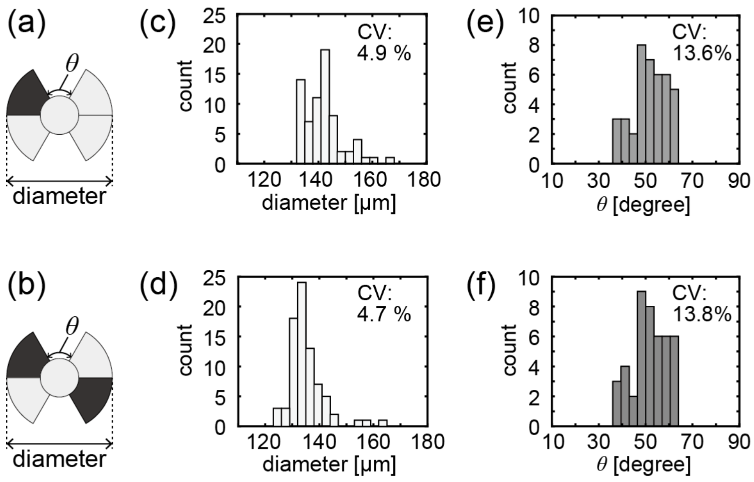

Figure S4. Measurements of size and shapes of the propeller-shaped micromotors. We measured the diameter and the angle θ of the propeller blades of the propeller-shaped micromotors with a single catalytic site (**a**) and double catalytic sites (**b**); (**c**,**d**) present the size distributions of the propeller-shaped micromotors with a single catalytic site and double catalytic sites, respectively. Mean diameters of the propeller-shaped micromotors were 141.9 μm (a single catalytic site) and 135.2 μm (double catalytic sites) with the coefficient of variation (C.V.) of ~5%. Thus, the sizes of the obtained propeller-shaped micromotors were highly monodisperse; (**e**,**f**) present the θ distributions of the propeller-shaped micromotors with a single catalytic site and double catalytic sites, respectively. In both cases, mean angle θ = 51.6° with the C.V. of ~10%. The results also suggest that the shapes of the propeller-shaped micromotors were relatively uniform.

Information for the Videos S1–S4

In all videos, the reproduction speed is ×1, and the frame size is 2133 × 1600 μm. Video S1: The motion of the propeller-shaped micromotors with a single catalytic site shown in Figure 2d. Video S2: The motion of the propeller-shaped micromotors with a single catalytic site shown in Figure 2e. Video S3: The motion of the propeller-shaped micromotors with double catalytic sites shown in Figure 3d. Video S4: The motion of the propeller-shaped micromotors with double catalytic sites shown in Figure 3e.
